# Tri-side-by-side stent deployment for hepatic hilar obstruction using fully covered self-expandable metal stents with a spiral-grooved outer surface

**DOI:** 10.1055/a-2888-0596

**Published:** 2026-06-24

**Authors:** Takeshi Ogura, Kimi Bessho, Junichi Nakamura, Nga Nguyen Trong, Hiroki Nishikawa

**Affiliations:** 1 Pancreatobiliary Advanced Medical Center 38588 Osaka Medical and Pharmaceutical University HospitalTakatsukiJapan; 2 Endoscopy Cente 38588 Osaka Medical and Pharmaceutical University HospitalTakatsukiJapan; 3 2nd Department of Internal Medicine 13010 Osaka Medical and Pharmaceutical UniversityTakatsukiOsakaJapan; 4 Department of GastroenterologyTrong Nam Cancer HospitalHà Noˆ̣iViet Nam

**Fig. 1:**
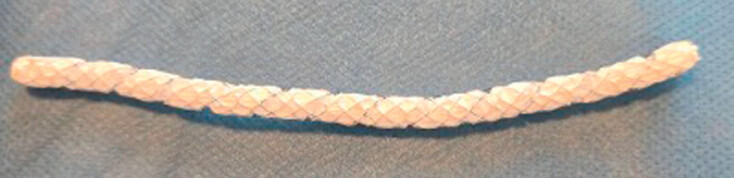
A fully covered self-expandable metal stent with a spiral-grooved outer surface (6-mm diameter, 6Fr stent delivery system, HIRZO Biliary Willow Stent; BCM Co., Ltd, Seoul, Korea).

In patietns with malignant hilar biliary obstruction (MHBO), stent deployment under endoscopic retrograde cholangiopancreatography (ERCP) can be considered a first-line treatment technique. 1​ 2​ 3​ 4​ ​5​ Recently, similar stent patency has been reported between uncovered self-expandable metal stents and inside plastic stents. To obtain longer stent patency, fully covered SEMS (FCSEMS) deployment using a side-by-side technique (SBS) might be an option. However, FCSEMS deployment for MHBO is indicated for selected patients because of side bile duct branch obstruction. To overcome this issue, a novel FCSEMS with a spiral-grooved outer surface (6-mm diameter, 12-cm length, 6Fr stent delivery system, HIRZO Biliary Willow Stent; BCM Co., Ltd, Seoul, Korea) has been developed ( Fig. 1 ). We describe herein technical tips for tri-SBS using this stent for high-grade MHBO.

**Fig. 2:**
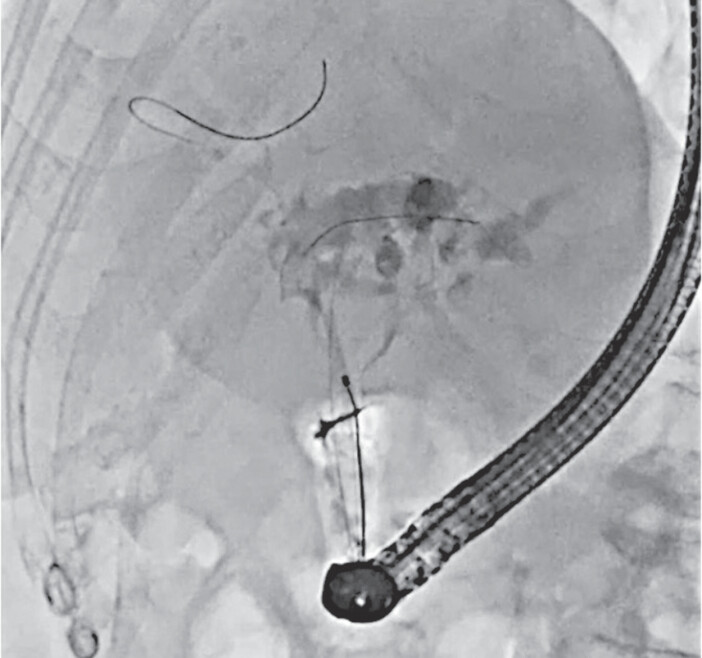
Guidewires are deployed at the left hepatic and posterior bile ducts.

**Fig. 3:**
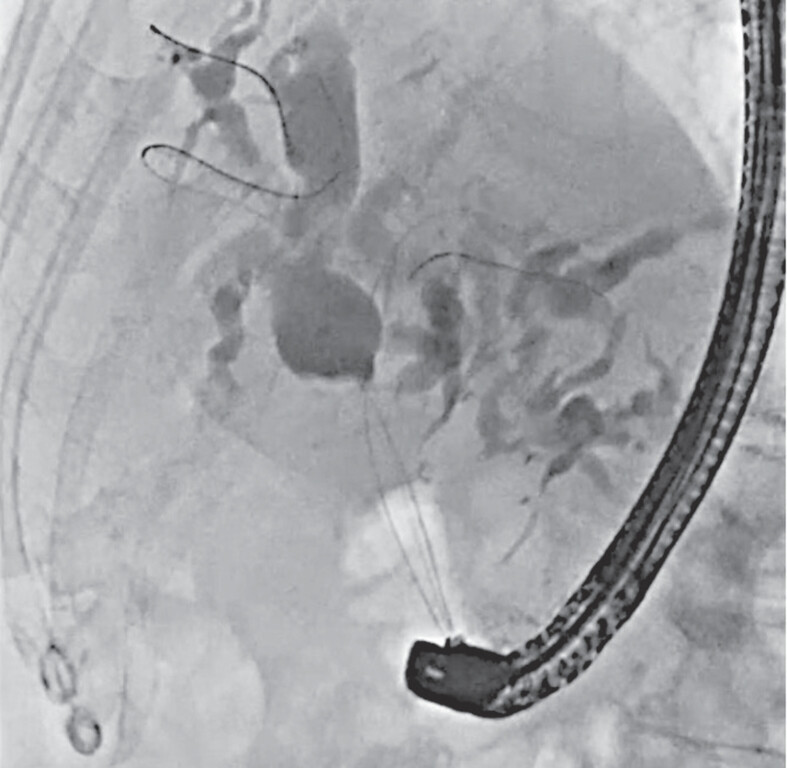
A guidewire is deployed at the anterior bile duct.

**Fig. 4:**
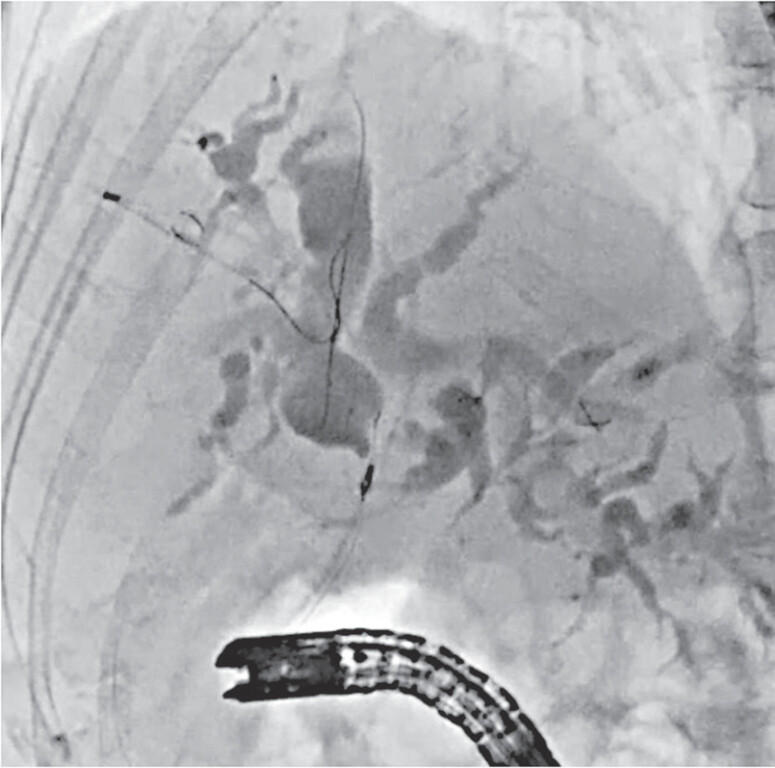
A fully covered self-expandable metal stent with a spiral-grooved outer surface is deployed at the left hepatic bile duct and the posterior bile duct using a side-by-side technique.

**Fig. 5:**
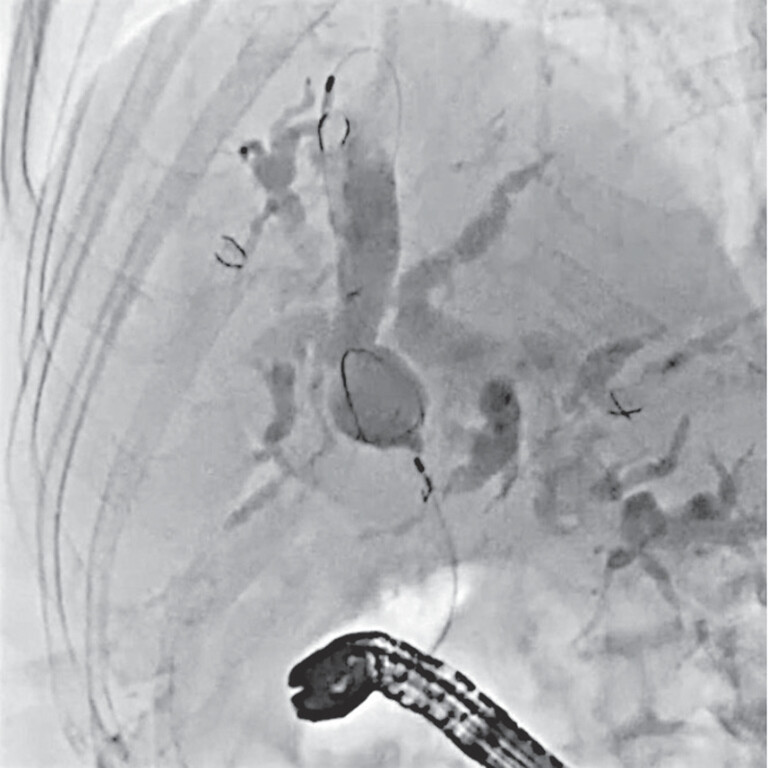
A stent delivery system for the fully covered self-expandable metal stent with a spiral-grooved outer surface can be smoothly inserted and successfully deployed into the anterior bile duct beside previously deployed metal stents.

A 68-year-old man was admitted due to obstructive jaundice. Choledochoduodenostomy was performed due to gallbladder and bile duct stones. Based on computed tomography and upper gastrointestinal endoscopy, hilar cholangiocarcinoma with invasion of the choledochoduodenostomy site was diagnosed. The patient underwent plastic stent deployment, but recurrent biliary obstruction was identified as a complication after 1 month. Re-intervention was therefore attempted. First, an ERCP catheter was inserted into the biliary tract and the guidewire was deployed into the left hepatic bile duct. Guidewires were subsequently deployed at the anterior and posterior bile ducts ( Fig. 2 ). Based on cholangiography, all bile ducts were obstructed ( Fig. 3 ). The FCSEMS with a spiral-grooved outer surface was deployed at the left bile duct, and also deployed at the posterior bile duct using SBS ( Fig. 4 ). The stent delivery system for a FCSEMS with a spiral-grooved outer surface was easily and smoothly inserted into the anterior bile duct beside previously deployment stents, and successfully deployed without any adverse events ( Fig. 5 ; Video 1 ). Computed tomography showed no bile duct branch obstruction. This patient received systematic chemotherapy, but died after 6 months. During this period, no stent dysfunction was observed.

**Video 1** Computed tomography after tri-side-by-side stent deployment using a fully covered self-expandable metal stent with spiral-grooved outer surface shows no bile duct branch obstruction by stents.


In conclusion, tri-SEMS deployment by SBS using the FCSEMS with a spiral-grooved outer surface may open a new window to MHBO drainage.

Endoscopy_UCTN_Code_TTT_1AR_2AZ
